# Reprocessing Possibilities of Poly(3-hydroxybutyrate-co-3-hydroxyvalerate)–Hemp Fiber Composites Regarding the Material and Product Quality

**DOI:** 10.3390/ma17010055

**Published:** 2023-12-22

**Authors:** Wiesław Frącz, Andrzej Pacana, Dominika Siwiec, Grzegorz Janowski, Łukasz Bąk

**Affiliations:** 1 Department of Materials Forming and Processing, Rzeszow University of Technology, Powstancow Warszawy 8, 35-959 Rzeszow, Poland; wf@prz.edu.pl (W.F.); gjan@prz.edu.pl (G.J.); lbak@prz.edu.pl (Ł.B.); 2 Department of Manufacturing Processes and Production Engineering, Rzeszow University of Technology, Powstancow Warszawy 8, 35-959 Rzeszow, Poland; d.siwiec@prz.edu.pl

**Keywords:** PHBV, biocomposites, injection molding, biocomposites reprocessing, recycling

## Abstract

An important issue addressed in research on the assessment of the quality of polymer products is the quality of the polymer material itself and, in accordance with the idea of waste-free management, the impact of its repeated processing on its properties and the quality of the products. In this work, a biocomposite, based on poly(3-hydroxybutyrate-co-3-hydroxyvalerate) (PHBV) with short hemp fibers, was obtained and repeatedly processed, which is a continuation of the research undertaken by the team in the field of this type of biocomposites. After subsequent stages of processing, the selected mechanical, processing and functional properties of the products were assessed. For this purpose, microscopic tests were carried out, mechanical properties were tested in static tensile and impact tests, viscosity curves were determined after subsequent processing cycles and changes in plastic pressure in the mold cavity were determined directly during processing. The results of the presented research confirm only a slight decrease in the mechanical properties of the produced type of biocomposite, even after it has been reprocessed five times, which gives extra weight to arguments for its commercialization as a substitute for petrochemical-based plastics. No significant changes were found in the used parameters and processing properties with the stages of processing, which allows for a predictable and stable manufacturing process using, for example, the injection molding process.

## 1. Introduction

Waste management is one of the most urgent challenges of modern society. In particular, the problem of plastic waste is becoming more and more urgent. Plastics are present in many areas of our lives and are an integral part of the modern economy. However, their durability and, in most cases, their lack of biodegradability, coupled with the growing production and consumption of plastics, have led to a growing ecological crisis. When these materials enter the environment, they remain there for dozens or even hundreds of years, contributing to soil and water pollution. Some plastics, especially those containing harmful chemicals, can lead to serious human health problems. For instance, phthalates and bisphenol A (BPA) can disrupt the body’s hormonal balance and cause various types of diseases. The lack of effective management of plastic waste results in the waste of mineral resources. Despite progress in the field of plastic recycling, many challenges remain. The lack of a uniform waste segregation and collection system, low recycling rates in some countries and the lack of appropriate processing technologies are serious problems that have prompted a search for new solutions [1,2,3,4].

One of the most important steps in solving the problem of plastic waste management is reducing the consumption of these materials. The introduction of innovative packaging, the promotion of a circular economy and consumer education are key in this context. Creating innovative recycling methods, such as the chemical decomposition of plastics into primary components, may be an important step forward [5,6,7]. Furthermore, the development of alternative biodegradable and compostable materials may contribute to solving the problem and finding a solution. Promoting sustainable materials, such as bioplastics, can lead to a reduced burden on the environment [8,9]. Among a fairly wide range of bioplastics, special attention should be paid to double green polymers, because they are of natural origin and are fully biodegradable. It is worth noting that their possibilities of use may be multiplied due to the continuous circulation of this type of materials and their derivatives in nature [10,11].

The circular economy is an economic model that aims to minimize the amount of waste by maximizing the use and reprocessing of resources. Unlike the traditional linear model, in which raw materials are consumed and discarded, the circular economy focuses on closing product life cycles, reducing the impact on the environment and using resources more efficiently [12]. With the growing problem of plastic pollution and the growing environmental awareness of society, the need to find sustainable alternatives to traditional plastics is becoming an increasingly pressing challenge. Polyhydroxyalkanoates (PHAs) are emerging as a revolutionary category of biodegradable polymers, potentially transforming the way we think about the production, consumption and disposal of materials [13].

Polyhydroxyalkanoates (PHAs) are a type of biodegradable polymer obtained from natural sources, such as bacteria, which use them as energy storage substances. In recent years, interest in these polymers has increased, due to their potential to replace traditional, non-biodegradable plastics. They are distinguished not only by their ability to degrade in the environment, but also by a variety of properties, which means they can be used in many areas, from packaging to medicine [14]. The structure of PHAs may vary depending on the source of the microorganisms which they are obtained from. Their structural diversity depends mainly on the type of monomers from which they are composed [15]. There are several key types, such as poly(3-hydroxybutyric acid) (PHB), poly(3-hydroxyvaleric acid) (PHV), and copolymers such as poly(3-hydroxybutyric acid-co-3-hydroxyvaleric acid) (PHBV). The PHA production process typically involves the use of bacteria. However, there is also a growing interest in the production of PHAs using plant organisms, which opens up new opportunities for the sustainable production of biodegradable plastics [16,17]. The PHA production process uses microorganisms with a high storage capacity and diverse biochemical processes, in order to increase the number of cycles of the “growth” and “starvation” phases [18,19]. It is also worth noting that the sterilization of reactors for the PHA production process is not necessary, and bacterial cultures are able to adapt to various additional waste raw materials [20]. The main advantage of PHA production is the ability to use real fermented waste as raw materials, such as agricultural or food industry by-products, which reduces the costs of substrate use [21,22]. PHAs exhibit a variety of properties that make them attractive for a variety of applications. Their biodegradability is a key element that enables their decomposition in natural conditions. Their biocompatibility makes them useful in the field of medicine, and their thermoplasticity allows them to be formed many times [23]. Despite promising prospects, the implementation of PHA materials is a challenge. Production costs and ethical issues related to the genetic modification of organisms require further research. Nevertheless, technological advances and society’s commitment towards sustainable alternatives will contribute to the development of this group of polymers. The prospect of lower production costs and growing ecological awareness opens the door for PHAs to become a key element in the global movement to counteract petrochemical plastic pollution [24,25].

Polyhydroxybutyrate-co-valerate (PHBV) is a type of biodegradable polymer that is a copolymer of poly(hydroxybutyrate) (PHB) and poly(hydroxyvalerate) (PHV). This combination of two different monomers introduces flexibility into the polymer structure, which makes PHBV more elastic than PHB alone [26]. Therefore, this material has a number of applications in various fields, such as the production of packaging, disposable products and biodegradable films. Broader commercial uses of this biopolymer are still difficult due to high production costs and a small difference between the melting point and the degradation temperature of this polymer, as well as low flexibility and quite high brittleness [27,28,29,30]. For this reason, further research plans of scientists include improving the mechanical properties and processing window of this biopolymer, as well as the possibility of producing composites based on PHBV [31,32]. It should be noted, however, that despite these advantages, PHBV production still encounters challenges related to, for example, production scaling. However, the development of this technology continues progressing, with the hope of finding more effective and economical production methods. The work by Guo, Stuckey and Murphy [33] presented the possibility of developing a PHBV production system without the use of fossil fuels. PHBV polymers have a slightly lower energy consumption during production per kg of polymer than petrochemical polymers. The current production processes and scale of PHBV production are still largely undeveloped, compared with the well-developed production of petrochemical polymers. It is forecast that further optimization of PHBV production technology and the expansion of its production scale may result in an improvement in the environmental condition. Additionally, the results of the work show that the use of renewable sources, instead of fossil electricity and heat resources required for the production of PHBV, will ensure the effective optimization of the process. These results confirm the view that, due to the expansion in the development of bioplastics production and changes in the sources of generation for the electricity and heat consumed, the bioplastics production industry can be independent from fossil fuel products.

One of the possible ways of commercializing ecological composite materials, especially PHBV, may be the use of natural fillers, e.g., fibrous fillers in biopolymer matrices. It is expected that their use will improve the mechanical properties of the manufactured composites while maintaining complete biodegradability and low production costs, compared with pure biopolymers [34]. Fibers of plant origin are cheaper than synthetic fibers, such as glass fiber. Of course, it should be noted that the prices of natural fibers also depend on geographical location, a very important aspect in terms of the availability of suitable natural fibers [35,36]. In Europe, the main emphasis is placed on the production of flax fibers and, to a lesser extent, hemp fibers, while in Asia, hemp, jute and kenaf fibers are more popular. Kenaf is commercially grown in the United States, while sisal is widely cultivated in tropical African countries, the West Indies and the Far East. The largest global production of plant fibers is bamboo and sugar cane stalks [36]. Many car construction and equipment elements are manufactured on the basis of composites based on thermoplastics and natural fibers. Door panels, seat backrests, trunk elements and upholstery are made of this type of composites [37]. The use of natural fiber composites is mainly due to their lower production costs, weight reduction, recyclability and marketing incentives in the era of environmental protection. Natural fibers, such as linen, hemp, cotton and jute, have long been used in the production of textiles and other products, but now their role is expanding to the area of plastics [38]. Adding natural fibers to polymers can provide a number of benefits. Firstly, there is the potential to significantly reduce the amount of plastic used, which directly translates into reduced waste. Moreover, natural fibers are biodegradable, which means that products containing them will decompose more easily after the end of the product’s life [39]. Natural fibers as fillers can also improve the mechanical properties of plastics. For example, the addition of hemp fibers to a polymer can increase its tensile strength and fracture resistance [40]. In addition, the variation in structure in natural fibers adds an aesthetic appearance to products, which is particularly attractive to consumers looking for more ecological options without sacrificing attractive design. However, introducing natural fibers into plastics is not without its challenges. The mixing and forming processes must be adjusted to obtain optimal mechanical properties. Moreover, quality control and the standardization of production processes become crucial to maintain product consistency [41].

An important issue is the possibility of developing modern biodegradable polymers of natural origin, such as PHBV bioplastic and hemp fiber filler. Currently the studies conducted [42,43,44,45] indicate an improvement in the properties of the obtained composites, compared with pure PHBV, after the addition of this filler. Another very important issue, that may significantly affect the possibilities of commercialization of this type of composites, is the assessment of the possibility of their multiple reprocessing (recycling) and the examination of the impact of subsequent reprocessing cycles on the functional, mechanical and processing properties of the obtained materials and a quality of products.

## 2. Materials and Methods

### 2.1. Research Materials

A copolymer of poly(3-hydroxybutyric acid) and poly(3-hydroxyvaleric acid) PHBV was used to produce the biocomposite, with the trade name ENMAT Y1000P NaturePlast (Mondeville, France), a specific weight of 1250 kg/m^3^ and a softening temperature in the range of 165 °C to 175 °C [46]. ENMAT Y1000P belongs to the group of polyhydroxyalkanoates (PHA). The share of PHV in the biopolymer used was 8%.

The fillers used were hemp fibers with a length (L) of approximately 1 mm, produced by EKOTEX company (Kowalowice, Poland) and surface-modified with a 10% sodium hydroxide solution (the fibers were etched to improve adhesion to the polymer matrix). Fibers with an approximate length to diameter ratio (L/d) of 10 were used. The fibers were used to reduce production costs and improve some mechanical, processing and functional properties, compared with pure biopolymers, while maintaining the ability to biodegrade [42,43,44,45]. These fibers are characterized by a cellulose content of approximately 68%, a hemicellulose content of approximately 15%, and a lignin and other ingredients content of approximately 10% [37,47].

The produced biocomposite contained 30% of the mass share of hemp fibers and 70% of the mass share of the polymer matrix. The fiber type and the mass fraction of the filler were chosen based on the results of previously conducted research, i.e., to improve the mechanical, processing and functional properties, compared with pure PHBV [42,43,44,45].

Due to the large number of produced biocomposite moldings and reprocessed series, markings were introduced for the series of tests, which are summarized in Table 1.

### 2.2. Production of Composite and Test Samples

The process of manufacturing the PHBV–hemp fiber biocomposite consisted of several stages. After mixing the hemp fiber with PHBV, the resulting mixture was dried in a Chemland DZ-2BC (Szczecin Stargard, Poland) laboratory dryer equipped with a vacuum pump. The drying process was carried out for 1 h at 90 °C.

The biocomposite of PHBV–hemp fiber was produced using an extrusion technological line, consisting of a single-screw extruder from ZAMAK EHP-25E (produced by ZAMAK Mercator company, Skawina, Poland) [48], a cooling bath and a granulator.

The PHBV–hemp fiber biocomposite was extruded at the extruder processing temperature profile shown in Table 2. The extrusion was carried out at a screw rotation speed of 100 rpm.

The last stage of production was the granulation of the produced material and, as a result, the obtaining of biocomposite granules. A Zamak granulator (produced by ZAMAK Mercator company, Skawina, Poland) was used, equipped with a cutting tool (mill), which allowed for the collection of granules with cylindrical geometry. The obtained granulate, before the next stage of the process, was dried in a laboratory dryer for 1 h at 90 °C.

During the injection molding process for the test samples, a BOY55E injection molding machine (produced by BOY Maschines Inc., Exton, PA, USA) with a Priamus system was used, allowing for the control and monitoring of the injection molding machine.

**Figure 1:**
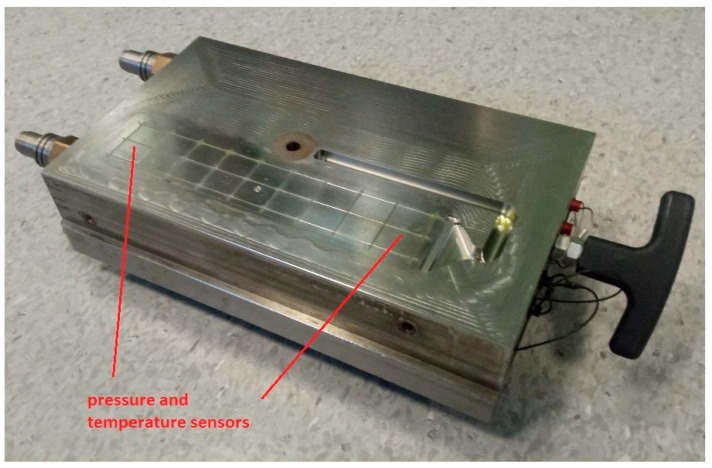
Injection mold with temperature and pressure sensors.

The injection mold was equipped with temperature and pressure sensors (Figure 1) as components of the Priamus system.

During the injection of the molded parts, the pressure in the mold cavity was measured using Priamus 6002B piezoelectric sensors [49] and the Priased 5080A (produced by Priamus System Technologies AG, Schaffhausen, Szwajcaria) four-channel amplifier integrated with them. The Fill Control software (version 1.0) used allowed for the recording of data from measurement channels for individual zones [50].

The temperature was measured using N-type thermoelectric sensors, which were mounted in the flow path at the same distance as the pressure sensors. The obtained measurements allowed the determination of the rheological characteristics of the composite and its subsequent processed batches.

In the first stage of the research, the samples with dog-bone geometry were produced from granules of the PHBV–hemp fiber biocomposite. The adjustable processing parameters of this material are presented in Table 3. During subsequent injection cycles, the viscosity values of the biocomposite, as a function of the shear rate and the profile of pressure changes, were recorded.

**Figure 2:**
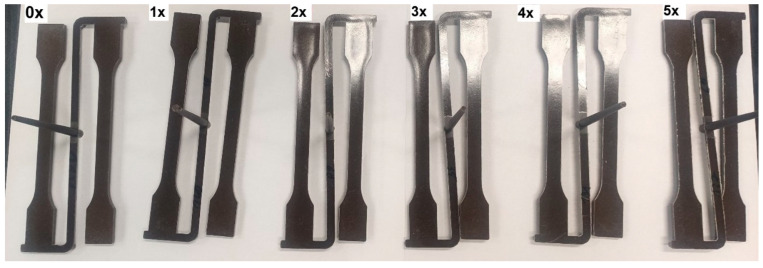
Samples from the original composite (0x) and subsequent reprocessing cycles (1x–5x).

After mechanical property tests, the manufactured molded pieces were ground using a Wanner C17 (Wertheim, Germany) plastics mill. The ground pieces were dried for 1 h at 90 °C, and then the samples with dog-bone geometry were injected again. Again, changes in cavity pressure and viscosity were measured as a function of shear rate. The procedure for subsequent processing of the tested biocomposite was carried out five times. During the processing and testing of subsequent series of biocomposite, the same adjustable parameters were used in the injection molding process. Correction of these parameters was not required, due to the fact that the molded pieces obtained in subsequent series were of good quality, in terms of their shape and dimensions (Figure 2). There were also no organoleptically visible effects of degradation of the biocomposite in the form of flashes, burns or underflows after subsequent series of reprocessing, which may confirm that the quality of the processed biocomposite after repeated processing does not significantly deteriorate, in terms of the possibility of another processing cycle.

### 2.3. Research Methods

**Uniaxial tensile test.**

The Zwick Z030 (produced by Zwick Roell, Ulm, Germany) testing machine was used to test the strength of the obtained composites. The uniaxial tensile test was carried out in accordance with the EN ISO 527-1 standard [51] for molded pieces with dog-bone geometry. Each series of samples consisted of seven molded pieces. Based on the obtained test results, the following were analyzed: the Young’s modulus (E), tensile strength (σ_M_) and the relative elongation at maximum tensile stress (ε_M_). The results were analyzed statistically; the following were determined: the arithmetic mean ($\bar{x}$), standard deviation (s) and coefficient of variation (V).

**Brinell hardness**

**Figure 3:**
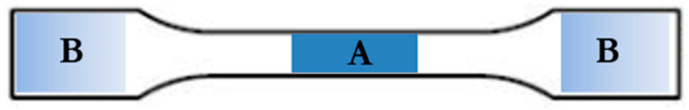
Areas of the sample for hardness testing.

The hardness assessment of the biocomposite was carried out using the Brinell method with the EN ISO 2039-1 standard [52] in two areas of the sample (Figure 3), i.e., in the measurement zone (zone A) and in the gripping part (zone B). A Zwick 3106 (produced by Zwick Roell, Ulm, Germany) hardness tester was used for this purpose. Each series of samples consisted of seven pieces.

**Impact tensile test**

In order to determine the impact tensile strength of biocomposites, tests were carried out in accordance with the EN ISO 8256 standard [53] using a CEAST 9050 pendulum hammer (produced by Instron Inc. Europe, Buckinghamshire, UK). The samples were cut from the ones for uniaxial tensile testing, in accordance with the requirements of the standard. The notch was made for entire sample packages. Each series included seven samples.

**Microstructure studies**

To visually assess the sample surfaces and fiber geometry, a Nikon MM-800 workshop (produced by Nikon Inc., Tokyo, Japan) microscope with E-MAX software was used. The dimensions measured were min. fifty fibers on the top layer of the molded piece, for the first and subsequent processed series. The measurements were performed for each sample in the same area of the molded piece. 

Microstructure tests were carried out using a HITACHI S-3400 scanning electron microscope (SEM) (produced by Hitachi Inc., Tokyo, Japan), based on specimens from the uniaxial tensile test.

**Shrinkage assessment**

The shrinkage of the molded parts with dog-bone geometry was tested partly based on the EN ISO 294-4 standard [54] The primary shrinkage was tested after approx. 3 h, and the secondary shrinkage was tested approx. 14 days after the molded pieces were manufactured by means of the injection molding process. The tests were performed for a series of seven samples.

**Rheological test**

The determination of the viscosity curves for the repeatedly injected biocomposite was done by recording the pressure and temperature, using appropriate sensors mounted in the injection mold cavity. The melted plastic is rheologically described by the Newtonian model. Its viscosity, in this case, can be calculated as [55]:

(1) $$\eta =\frac{\tau }{\gamma }$$

where:

η—viscosity,

*τ*—shear stress,

and *γ*—shear rate.

Calculation of the shear stress and shear rate is possible thanks to the knowledge of the geometry of the injection mold cavity and the pressure values in two different places along the flow path of the polymer material. For a rectangular mold cavity cross-section:

(2) $$\gamma =\frac{3*\dot{Q}}{4*k^{2}*z}$$

(3) $$\tau =\frac{∆p*k}{W}$$

where:

$\dot{Q}$—flow rate,

k—cavity height,

z—cavity width,

∆p—pressure difference between pressure sensors,

and W—distance between pressure sensors.

Knowing the relationships for shear rate (2) and shear stress (3), Equation (1), used to determine the viscosity, can be written as:

(4) $$\eta =\frac{\tau }{\gamma }=\frac{\frac{∆p\;*\;k}{W}}{\frac{3\;*\;\dot{Q}}{4\;*\;k^{2}\;*\;z}}=\frac{4\;*\;k^{3}\;*\;z\;*\;∆p}{3\;*\;\dot{Q}\;*\;W}$$

Viscosity curves were determined for newly produced (0x) and multi-reprocessed (1x, 2x, 3x, 4x, 5x) PHBV–hemp fiber biocomposites. Viscosity measurements were made for various temperatures of the melt (180 °C, 185 °C and 190 °C), at injection rates ranging from 10 to 70 cm^3^/s.

## 3. Results and Discussion 

**Figure 4:**
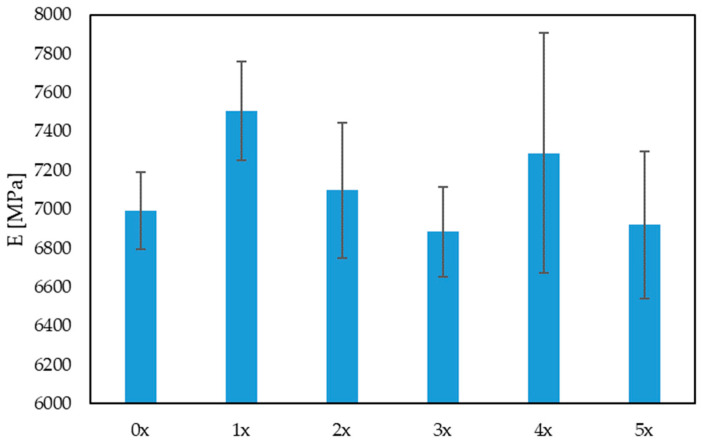
Young’s modulus for multi-reprocessed biocomposites.

**Figure 5:**
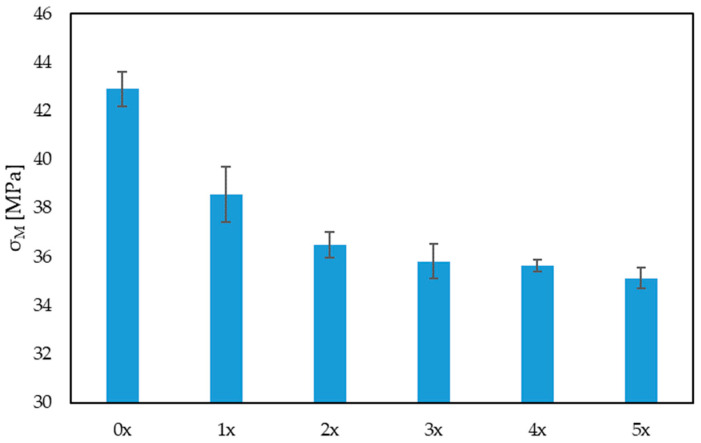
Tensile strength for multi-reprocessed biocomposites.

The results of the uniaxial tensile test were analyzed, taking into account the Young’s modulus, tensile strength and elongation of the samples. When analyzing the values of the Young’s modulus (Figure 4), there was no visible trend of a decrease or an increase in its value. However, it was observed that, with the next reprocessing cycle, the dispersion of the results increased, which may indicate a deteriorating homogeneity of the biocomposite. In turn, in the case of tensile strength (Figure 5), there was a visible trend associated with a decrease in its value after subsequent reprocessing cycles, by up to 18% (for the 5x biocomposite), compared with the originally produced biocomposite (0x). There was also a noticeable decrease in the maximum elongation of the sample, by approximately 13% for the 5x biocomposite, compared with the 0x biocomposite. When analyzing these three properties, a significant impact of reprocessing on the mechanical properties of the products can be noted.

**Figure 6:**
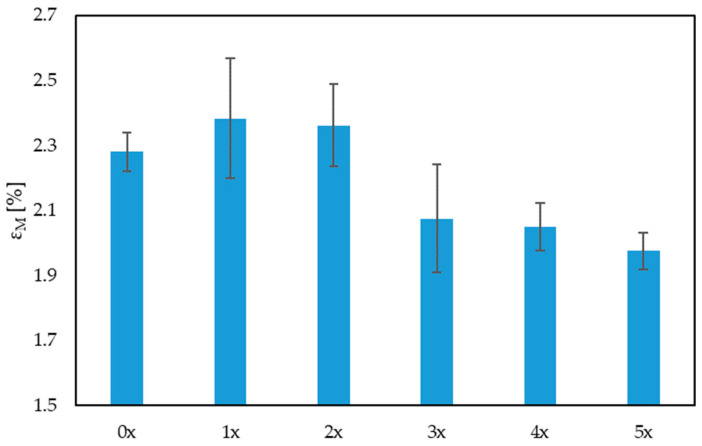
Relative elongation at the maximum tensile stress for multi-reprocessed biocomposites.

When adding natural fibers to the polymer matrix, one can increase the tensile strength of the composite. These fibers act as reinforcement, improving the load-bearing capacity of the composite along the tensile direction [44,56]. After repeated cycles of processing, the fibers become mechanically shortened, and carry loads in their longitudinal direction to a lesser extent, which reduces their tensile strength. The reduction in sample elongation (Figure 6) may be related to the thermal load history of the biocomposite, which becomes less and less flexible after each reprocessing. Additionally, multiple reprocessing can affect the molecular structure of the polymer, which in turn affects its mechanical properties. This may lead to a loss of elasticity and is manifested by a reduced ability to deform before fracture [57,58].

**Figure 7:**
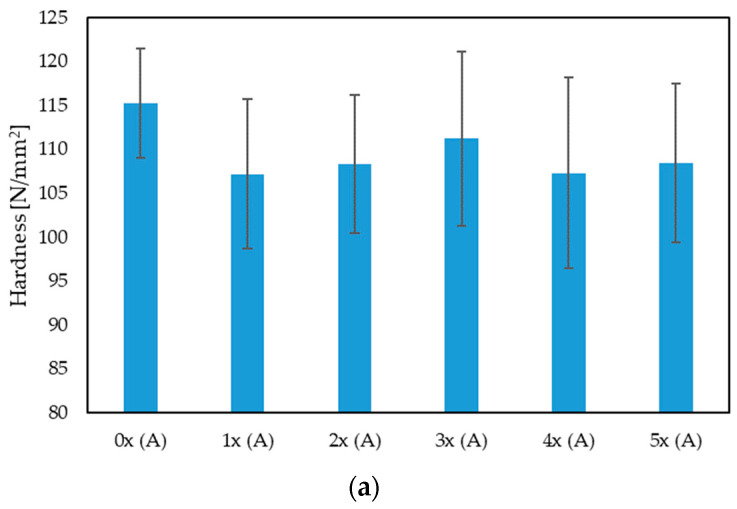

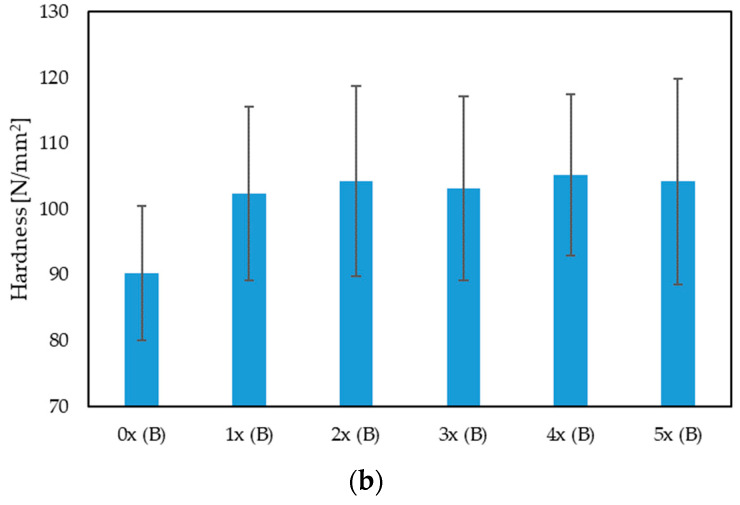
Hardness for multi-reprocessed biocomposites, in areas A (**a**) and B (**b**) of the molding.

The results of the Brinell hardness tests indicate a slight decrease after repeated processing of the biocomposite in area A of the sample, i.e., in the narrowing (Figure 7a). The decrease in value is approximately 6% for the 5-fold recycled biocomposite (5x), compared with the originally produced one (0x), but this result is within the standard deviation. In the case of hardness tests in area B (Figure 7b), there is a noticeable increase in hardness by approximately 15% for the 5x biocomposite, compared with 0x. Basically, it can be noted that, for the initially produced biocomposite (0x), there is a significant difference in hardness in the area A (by approx. 28%) compared with area B. A significant influence of the geometry and fiber distribution in the polymer matrix is visible here. The fibers in the area of the measuring part of the sample are oriented along the length of the sample and are parallel to each other, which results from the constant geometry of the mold cavity. This results, among others, in an even distribution of fibers in layers throughout the thickness of the molded part, including its surface layer, and a significant improvement in its mechanical properties. In turn, in area B, i.e., the gripping part of the sample, the fibers are distributed more chaotically, due to the change in the geometry of the forming cavity. A similar trend is confirmed in publication [44], where the influence of adding hemp, wood and linen filler (15% by mass) on the improvement in hardness in relation to pure PHBV was examined—a significant improvement in hardness was found after adding each of the fibrous fillers in areas A and B. A similar trend was also noted here, i.e., in area A the hardness was significantly higher than in area B. From the tests carried out for the composite reprocessed five times, it can be noted that the hardness values in both areas A and B are similar to each other. This fact may be due to the shortening of the fiber length by the repeated grinding of the samples. As a result of the shortening of their length, the fibers are characterized by a lower aspect ratio (L/d) and lose their mechanical properties as a typical reinforcement of the polymer matrix.

Testing the hardness of plastics using the Brinell method is an important tool in the field of materials engineering, allowing for the assessment and control of the quality of materials at various stages of the production process and during operation. In the context of polymer biocomposites, this test is important because it allows one to determine how this material behaves under load, how easily it is deformed and what its mechanical properties are [59]. Hardness test results can be used to evaluate the quality, strength and applications of a given plastic. In the context of the quality of materials evaluation, hardness is an important parameter when assessing the quality of plastic products. Materials with higher hardness are usually more durable and resistant to abrasion, which is crucial for many applications, such as machine components, tools or structural elements [60,61]. Based on the results obtained (Figure 7), in this context, changes in hardness after repeated reprocessing cycles are small, and repeated reprocessing of the biocomposite itself may result in uniform hardness in various areas of the molded product.

**Figure 8:**
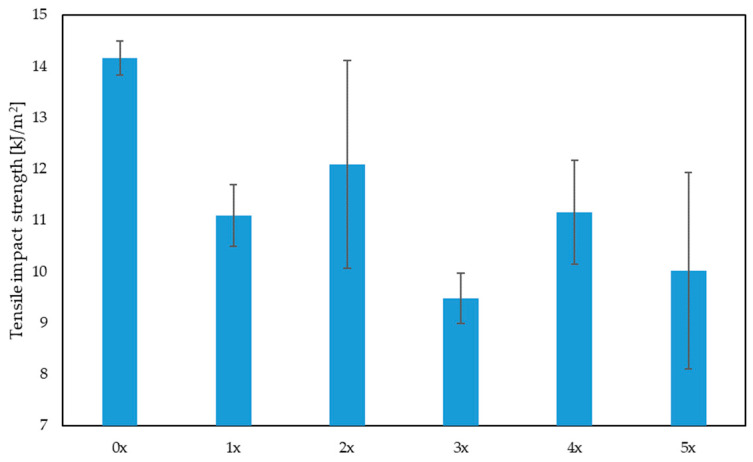
Impact tensile strength for biocomposites depending on the multiplicity of processing.

When analyzing the results of the impact tensile strength (Figure 8), a significant decrease in this parameter can be noted after repeated cycles of reprocessing. The impact tensile strength of the 5-times processed biocomposite (5x) is approximately 29% lower than the original biocomposite (0x). It is also possible to notice a significant increase in the dispersion of test results after subsequent reprocessing attempts, which proves a greater diversity of sample properties within the tested group. The fibers embedded in the polymer matrix of the originally processed composite had a relatively constant length of approximately 1 mm. After multiple processing cycles, some fibers were shortened as a result of the grinding of the moldings, which varied the length of the fibers and, consequently, influenced the differences in the impact tensile strength of the reprocessed samples.

The influence of the impact strength of biocomposites with fiber filler on the quality of products may be significant, especially in the context of products that are exposed to dynamic loads, such as impacts or vibrations. Fiber fillers such as plant-derived fibers can improve the impact strength of biocomposites [44]. However, based on the impact tensile results (Figure 8), it can be concluded that the mere presence of fibers does not automatically guarantee an increase in impact strength. The impact strength of a biocomposite may be influenced by many factors, such as the type of fibers, their length, their orientation, their quantity and the repeated processing of the biocomposite [62].

Fiber fillers have a significant impact on the longitudinal shrinkage of materials. The fibers reinforce the structure, which may reduce the tendency of longitudinal shrinkage. Fibers of appropriate length and distribution can improve the elasticity of the material, which translates into a reduced risk of deformation under the influence of longitudinal stresses. Fibrous fillers also play a key role in controlling transverse shrinkage. Fibers, dispersed evenly in the material, can prevent excessive transverse shrinkage, which is especially important in the case of materials subject to changing environmental conditions. It is also worth raising the issue of the impact of natural fibers on shrinkage through the thickness of the product. Properly selected fibers can increase the density of the material, which translates into greater thickness. However, there is a subtle balance between the number of fibers and the preservation of mechanical properties. Excess fibers may lead to the compaction of the material, which may consequently reduce the thickness of the products [63,64,65].

**Figure 9:**
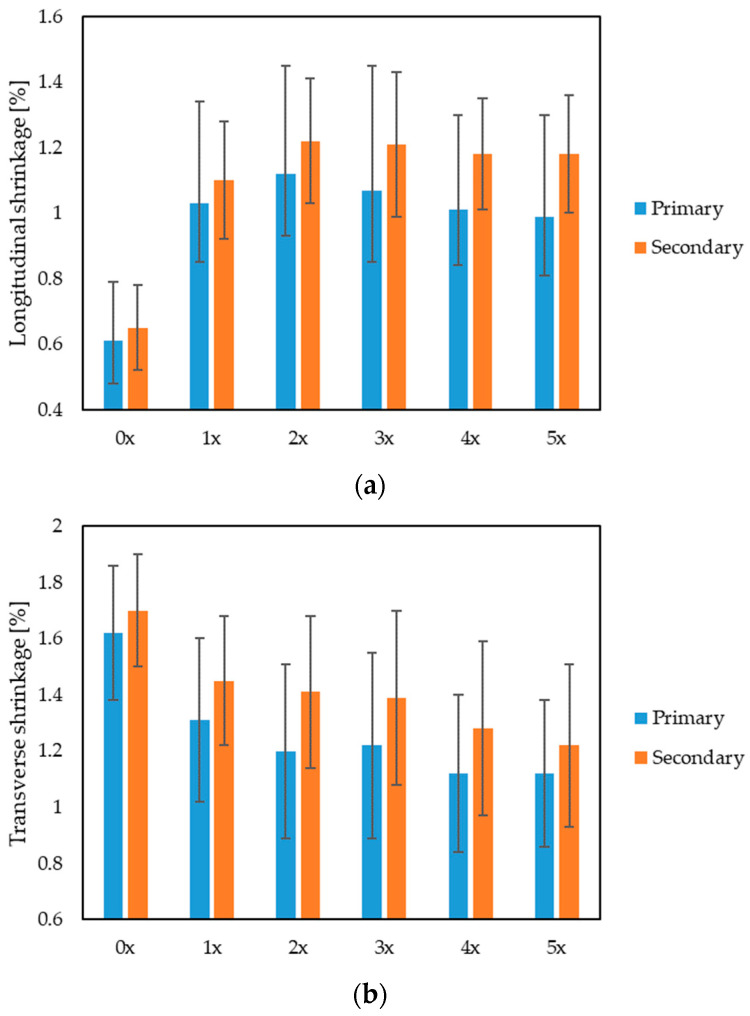

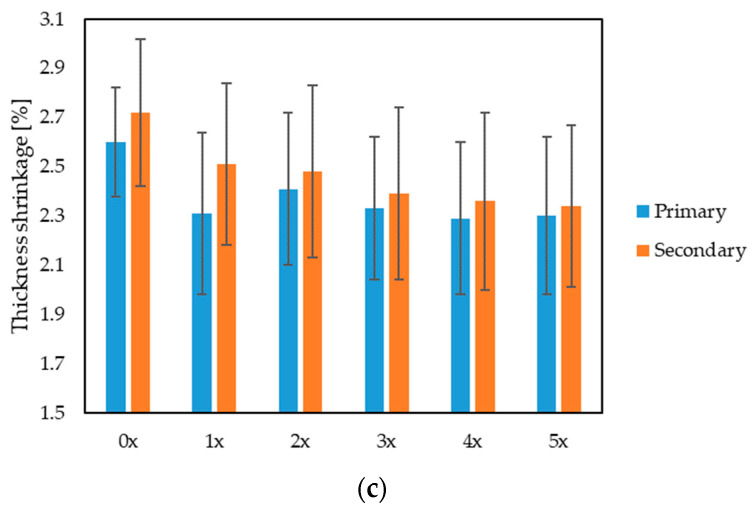
Primary and secondary shrinkage of injection molded parts: (**a**) longitudinal, (**b**) transverse and (**c**) in thickness, for multi-reprocessed biocomposites and the primary biocomposite.

When analyzing the values of longitudinal shrinkage (Figure 9a), a significant increase in the value of this parameter can be found with subsequent reprocessing cycles. The fibers shorten with each subsequent grinding of the moldings, which reduces the importance of this filler as reinforcement in the longitudinal direction. The value of secondary longitudinal shrinkage increased by almost 82% for the 5x biocomposite, compared with the original 0x. In turn, the value of secondary transverse shrinkage (Figure 9b) was reduced by approximately 28% for the 5x biocomposite, compared with 0x. There was also a decrease in the secondary shrinkage in thickness (Figure 9c) by approximately 14% for the 5-times reprocessed biocomposite, compared with the original one. The reduction in shrinkage in these directions may be caused by the fragmentation of the hemp fibers after reprocessing, which are distributed throughout the entire volume of the melt, and not mainly in the longitudinal direction, as was the case with the originally processed biocomposite. 

Importantly, variable parameters characterizing the fibers after subsequent series of reprocessing were the length (L) and diameter (d) of the fibers, and, consequently, the so-called aspect ratio: L/d. This coefficient is important in the processing of composites filled with short fibers because, after exceeding the critical length (above the critical L/d value), the fibers do not function as reinforcement. They no longer improve the properties of composites, especially in the direction of loads acting along the fiber axis. A too-small contact surface of the fiber with the polymer matrix makes them crack in the middle of their length under the influence of stress [66,67,68].

**Figure 10:**
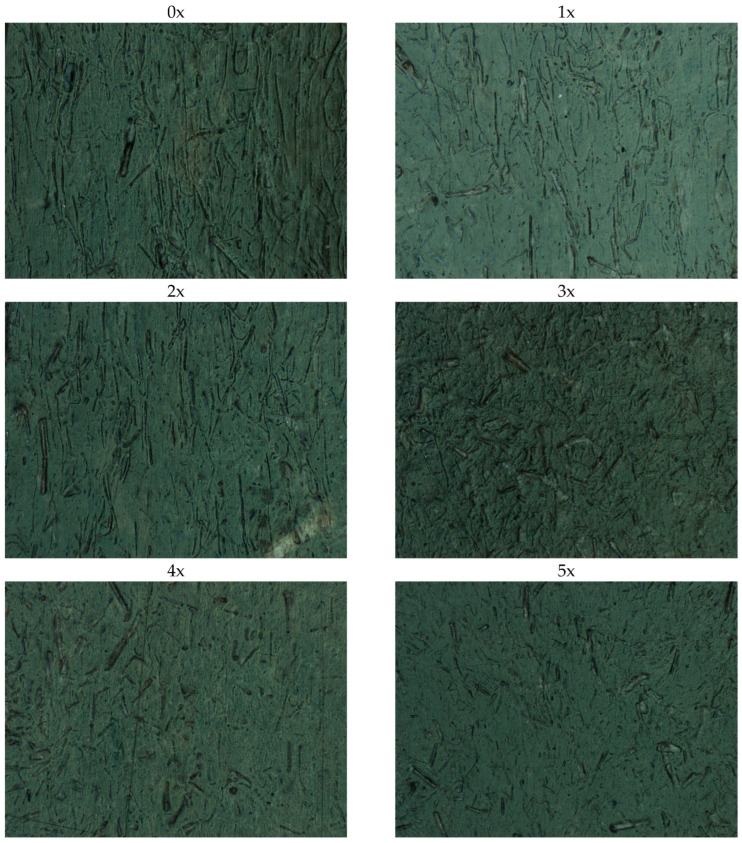
Photographs of fibers on the surface top layer of the molded piece for subsequent biocomposite reprocessing cycles (50× magnification).

When analyzing the lengths of the fibers presented in Table 4 and the photograph (Figure 10), it can be noticed that, for each processed series of the biocomposite, the fibers are shortened, and, after the final fifth processing cycle (5x), the hemp fibers were approximately 41% shorter in length than the fibers in the originally manufactured moldings (0x). There was no significant change in fiber diameter. As a consequence, the fiber aspect ratio decreased by approximately 42%. This reduction in the value of the L/d parameter results in, among others, the deterioration of the mechanical properties and the increased shrinkage of the molded parts, as evidenced by the results obtained. In turn, referring to the results regarding the rheological characteristics of the material, obtained experimentally during injection, the shortening of the length of the fibers reduces the viscosity of the flowing material (due to lower flow resistance, as the fibers are shorter after each injection cycle) and the occurrence of lower pressure values in the mold cavity. It is noticeable that, with each subsequent reprocessing cycle, the fibers are less and less oriented in a specific direction. In addition, an interesting phenomenon noted during fiber length measurements is the fact that the fibers are less and less visible on the surface layer of the molded part, which may indicate that they tend to stick together in the core of the molded piece.

**Figure 11:**
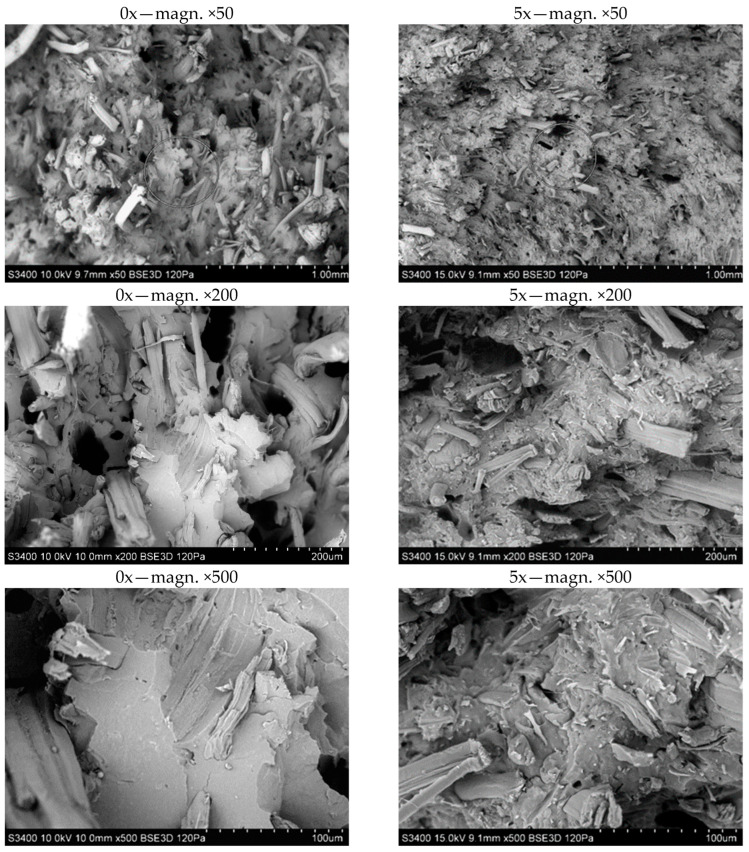
SEM photographs of the fracture surfaces of biocomposite samples for 0x and 5x biocomposites.

The fracture structure of the samples after the uniaxial tensile test is shown in Figure 11. SEM photographs were taken for samples originally produced (0x) and samples processed five times (5x). Photographs taken for the extreme conditions show increasing fiber fragmentation with the number of reprocessing cycles. The fibers also thin, and the polymer matrix shows signs of heat stress. The photos also indicate a good, uniform distribution of the fibers in the matrix. It can also be observed that the homogenization of fiber distribution in the matrix increases with the number of processing times.

**Figure 12:**
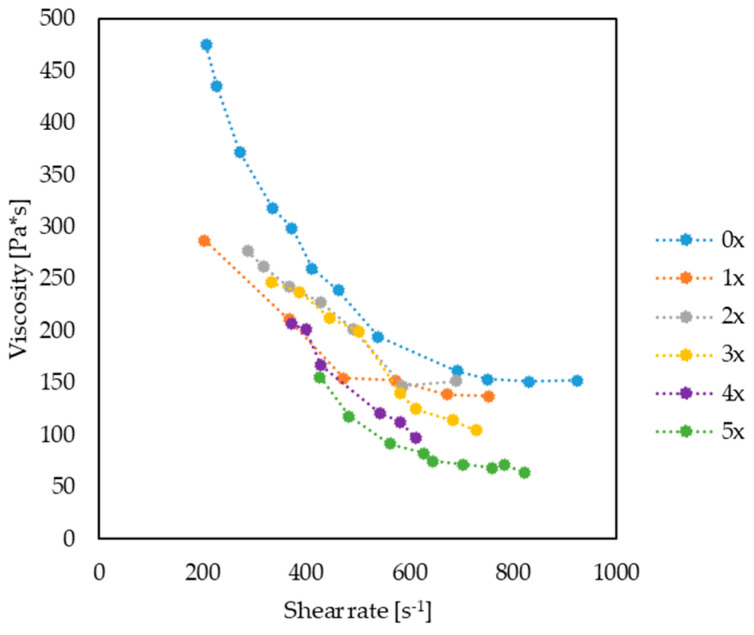
Examples of viscosity curves for biocomposites: 0x, 1x, 2x, 3x, 4x and 5x, at a temperature of 185 °C, obtained based on injection molding tests.

**Figure 13:**
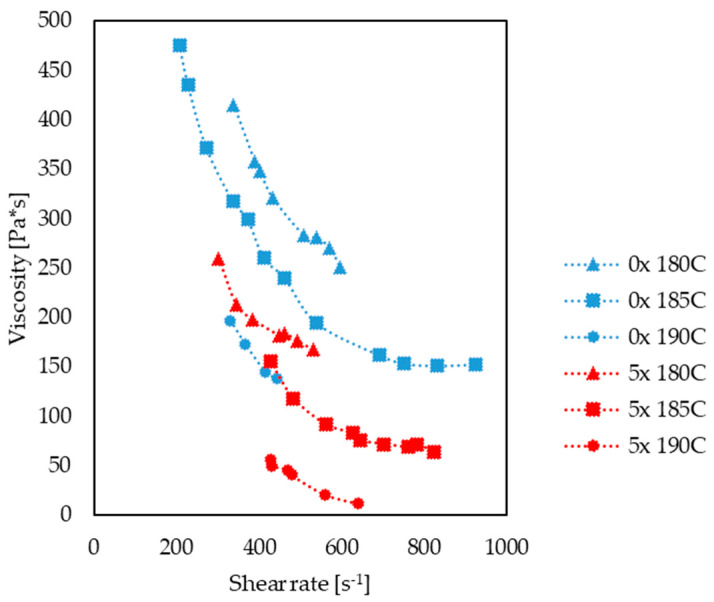
Comparison of viscosity curves for 0x and 5x biocomposites at temperatures of 180 °C, 185 °C and 190 °C.

The processing properties of the PHBV–hemp fiber biocomposites were determined for subsequent processing cycles (0x, 1x, 2x, 3x, 4x, 5x), during their injection into a special injection mold, by determining the viscosity curve. For each biocomposite, viscosity measurements were performed at various temperature of the melt plastic (180 °C, 185 °C and 190 °C) and at various injection speeds, ranging from 10 to 70 cm^3^/s. The experimentally determined changes in the biocomposite viscosity in the injection mold cavity are shown, for example, in Figure 12. It was noted that, with increasing temperatures and reprocessing cycles, lower viscosity values were observed for the same shear rates. This relationship can be observed in particular when comparing the determined viscosity curve for the 0x and 5x biocomposites (Figure 13).

It should be noted that viscosity curves were experimentally obtained in narrow shear rate ranges. Very often, in practice and in scientific work, special rheological models are used to extrapolate viscosity values to shear rate areas for which no experimental tests have been carried out [55,69,70]. The experimentally obtained viscosity curves were extrapolated using the Cross–WLF (Cross-Williams–Landel–Ferry) model [71,72]:

(5) $$\eta (\gamma ,T,p)=\frac{\eta_{0}(T,p)}{1+{\left(\frac{\eta_{0}\;*\;\gamma }{\tau^{*}}\right)}^{1-n_{c}}}$$

where:

η—viscosity,

T—temperature,

p—pressure,

$n_{c}$ i $\tau^{*}$—Cross–WLF model constants,

and $n_{c}$—zero viscosity.

The change in zero viscosity as a function of temperature can be written as:

(6) $$\eta_{0}\left(T,p\right)=D_{1}*exp\left[-\frac{A_{1}*(T-T_{g})}{A_{2}+(T-T_{g})}\right]$$

where:

(7) $$T_{g}\left(p\right)=D_{2}+D_{3}*p$$

(8) $$A_{2}=A_{3}+D_{3}*p$$

where:

$T_{g}$—glass transition temperature,

and $D_{1},D_{2},D_{3},A_{1},A_{2},A_{3}$—Cross—WLF model constants.

**Figure 14:**
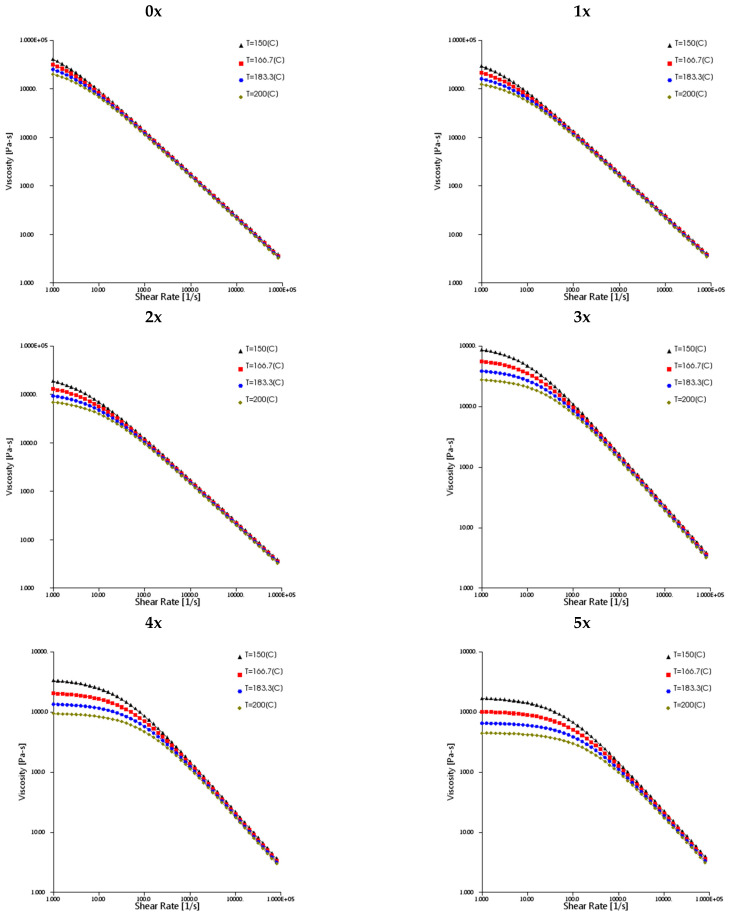
Viscosity curves calculated using Autodesk Moldflow Insight 2021 software for biocomposites processed multiple times (0x, 1x, 2x, 3x, 4x and 5x).

For the original and subsequently processed biocomposites, based on experimentally determined data, the values of the Cross–WLF model parameters necessary to determine viscosity curves in the shear rate range of 10^1^–10^6^ s^−1^ were determined by means of the Data Fit 9.0 software (Table 5). Viscosity curves, calculated using the Autodesk Moldflow Insight 2021 software (based on experimental data), are presented in Figure 14.

**Figure 15:**
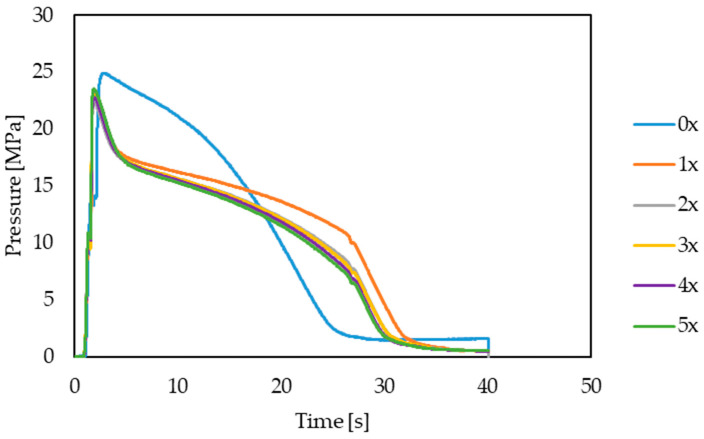
Representative pressure profiles in the mold cavity for subsequent biocomposite processing cycles.

Also in the context of viscosity changes, the impact of decreasing the viscosity of the biocomposite in subsequent processing cycles on the injection process itself was analyzed. Using the PRIAMUS system, the profiles of pressure changes in the molding cavity were recorded for subsequent cycles of the processed biocomposite. It was noted (Figure 15) that, with subsequent cycles, there was a noticeable decrease in the maximum pressure values in the mold cavity at similar shear rate values. A flowing stream of plastic with increasingly lower viscosity causes lower flow resistance, which leads to lower pressure values. This is important when controlling the quality of the injection molding process. Obtaining lower pressures in the mold cavity may result in the stabilization of the injection process and less exploitation of the injection molding machine and injection mold [73,74].

## 4. Summary and Conclusions

The impact of multi-reprocessing of biocomposites with hemp fibers on the quality of the injection products can be quite complex and depends on many factors. Repeated processing may lead to the gradual degradation of the fibers in the biocomposite. This, in turn, may affect the mechanical properties of the composite. Long-term processing may result in fibers breaking and shortening. The results obtained in the uniaxial tensile test indicate a deterioration of the mechanical properties in terms of tensile strength by up to approx. 18% and in terms of the elongation of the sample by approx. 13%, compared with the originally produced biocomposite. A similar trend was also noted in the context of impact tensile strength, where a decrease of approximately 29% in the value of this parameter was noted after the fifth reprocessing. In turn, when analyzing the hardness results in the two tested measurement areas, it is possible to note a decrease in hardness in the narrowing part of the sample by approximately 6%, where this decrease is within the standard deviation. In turn, in the case of measurements of the gripping part of the sample, an increase in hardness of approximately 15% was noted, which may be due to the fact that the shortened fibers are distributed more evenly in that part of the mold cavity, where the directionality of the flow of the material changes.

Taking into account the quality of the products in terms of their shape and dimensional accuracy, it was found that, with each reprocessing cycle, the longitudinal shrinkage of the product increased, which was the result of shortening of the fibers during the grinding of the molded pieces. With subsequent reprocessing cycles, the length of the fibers decreased, each time by an average of approx. 9–21%, with the largest decrease recorded for the first cycle (1x). This corresponds to the largest change in the value of longitudinal shrinkage, i.e., as much as 69%, and approx. 15% in the case of transverse shrinkage, also for the first cycle. For the second cycle, negligible changes in transverse shrinkage (approx. 2%) and slight changes in longitudinal shrinkage (approx. 10%) were recorded. Subsequent processing cycles do not bring significant changes, both in terms of fiber length and shrinkage value. Much shorter, fragmented fibers only have a small impact on the shrinkage of the polymer matrix. The observed decrease in the value of transverse and thickness shrinkage is most likely the result of the orientation of the shortened fibers not only in the longitudinal direction (as was the case with the originally processed biocomposite), but in the entire volume of the polymer matrix. The described phenomena, regarding changes in shrinkage, may be confirmed by tests of the composite structure on the surface layer of the molded part, which show that the fibers have actually shortened.

A very important issue in the context of multi-reprocessing of the PHBV–hemp fiber biocomposite are changes in the viscosity of the material, which require adjustments to the adjustable parameters of the processing process. The Priamus system was used in the research. The results indicate a general trend that the viscosity of this material decreases with the number of times the PHBV–hemp fiber biocomposite is processed. This is confirmed by the recorded pressure changes in the molding cavity—there are lower maximum pressure values are obtained after repeated processing. The recorded rheological behavior of the biocomposite may be due to the fact that the fibers, after shortening, generate a lower flow resistance while the plastic flows, which reduces the viscosity and also reduces the length of the polymer chains.

The impact of reprocessing biocomposites with natural fibers on the quality of injected products is an important issue in the fields of materials engineering and industry. Biocomposites, which are a combination of natural fillers and biopolymers, are becoming more and more popular due to their environmental friendliness. However, the repeated processing of these materials may generate challenges related to the quality of the final products. The first key aspect is the degradation of natural fibers due to repeated processing. Natural fibers, such as hemp fibers, may be shortened as a result of mechanical and thermal processes. As a result, their tensile strength and elasticity (as a result of thermal degradation) may decrease, which is an important factor affecting the mechanical properties of the biocomposite.

Another quality feature of injection molded products is dimensional stability. The impact of repeated processing on the structure of the tested biocomposite may lead to undesirable changes in the dimensions of the injected products. Therefore, it is necessary to monitor these changes and adjust the injection process to minimize the impact on dimensional accuracy. The surface quality of products injected from tested biocomposites subject to repeated processing also deserves attention. Possible changes in the structure of the tested material may affect the appearance and smoothness of the surface, which is especially important in the case of products with high aesthetic requirements. 

Ultimately, the question of the recyclability of biocomposites is becoming more and more important. Reprocessing must be consistent with the principles of sustainable development, and possible changes in the structure of materials should be taken into account in recycling processes too. The reprocessing of biocomposites may lead to changes in their rheological properties. An increase in the number of reprocessing cycles can affect the viscosity, the ability of the fibers to disperse in the polymer matrix and the ability of the biocomposite to flow during injection. A good understanding of the impact of repeated processing on the processing capabilities and rheology of biocomposites is essential to improve production processes, minimize material losses and achieve the optimal quality of molded products. Research on the impact of the reprocessing of biocomposites with natural fibers on the quality of injection molded products is of fundamental importance for the further development of this promising field of materials engineering.

It is necessary to constantly improve manufacturing processes, monitor changes in the properties of processing materials and search for innovative solutions aimed at minimizing the negative impact on the quality of final products. In this way, biocomposites with natural fibers can become an even more competitive alternative to traditional plastics, combining ecological benefits with high quality products.

## Figures and Tables

**Table 1:** List of produced and reprocessed series of biocomposites.

Designation	Biocomposite/Multiple Processing
0x	starting/primary material
1x	reprocessed
2x	twice processed
3x	processed three times
4x	processed four times
5x	processed five times

**Table 2:** Temperatures of the extruder heating zones.

Set Temperature [°C]			
Head	Zone 3	Zone 2	Zone 1
175	170	160	150

**Table 3:** Adjustable processing parameters for injection molding of the biocomposite.

Parameter	Value
Mold temperature [°C]	85
Melt temperature [°C]	185
Cooling time [s]	25
Packing time [s]	25
Packing pressure [MPa]	30
Flow rate [cm^3^/s]	35

**Table 4:** Average length L, diameter d and L/d aspect ratio for 50 fibers in the biocomposite after subsequent processing cycles.

Average Values from the Measurements of 50 Fibers			
Biocomposite	L [mm]	d [mm]	Aspect Ratio L/d
0x	0.991	0.113	8.770
1x	0.782	0.110	7.109
2x	0.714	0.113	6.319
3x	0.604	0.105	5.752
4x	0.493	0.108	4.563
5x	0.403	0.109	3.696

**Table 5:** Parameters of the Cross–WLF model of multi-reprocessed biocomposites.

	Biocomposite Processing Multiplicity					
Parameter	0x	2x	3x	4x	5x	6x
**n_c_ [-]**	0.1220	0.1291	0.1312	0.1395	0.1425	0.1587
τ* [Pa]	74,657.9	79,657.9	80,013.3	85,237.0	92,075.0	98,762.0
D_1_ [Pa*s]	1.78 × 10^11^	1.60 × 10^11^	1.40 × 10^11^	1.10 × 10^11^	9.00 × 10^10^	8.00 × 10^10^
**D_2_ [K]**	282.25	282.25	282.25	282.25	282.25	282.25
**D_3_ [K/Pa]**	0	0	0	0	0	0
**A_1_ [-]**	19.87	20.54	21.22	22.17	23.33	24.13
**A_2_ [K]**	51.6	51.6	51.6	51.6	51.6	51.6
**Determination ceofficient R^2^ [-]**	0.1220	0.1291	0.1312	0.1395	0.1425	0.1587

## Data Availability

The data presented in this study are available on request from the corresponding author.
